# Diagnostic performance of prediction models for extraprostatic extension in prostate cancer: a systematic review and meta-analysis

**DOI:** 10.1186/s13244-023-01486-7

**Published:** 2023-08-22

**Authors:** MeiLin Zhu, JiaHao Gao, Fang Han, LongLin Yin, LuShun Zhang, Yong Yang, JiaWen Zhang

**Affiliations:** 1 Department of Radiology, Huashan Hospital, Fudan University, Shanghai, 200040 China; 2 Department of Radiology, Sichuan Provincial People’s Hospital, University of Electronic Science and Technology of China, Chengdu, 610072 China; 3 Department of Pathology and Pathophysiology, Chengdu Medical College, Development and Regeneration Key Laboratory of Sichuan Province, Chengdu, 610500 China; 4 School of Big Health & Intelligent Engineering, Chengdu Medical College, Chengdu, 610500 China

**Keywords:** Prostatic neoplasms, Extraprostatic extension, Nomograms, Magnetic resonance imaging, Meta-analysis

## Abstract

**Purpose:**

In recent decades, diverse nomograms have been proposed to predict extraprostatic extension (EPE) in prostate cancer (PCa). We aimed to systematically evaluate the accuracy of MRI-inclusive nomograms and traditional clinical nomograms in predicting EPE in PCa. The purpose of this meta-analysis is to provide baseline summative and comparative estimates for future study designs.

**Materials and methods:**

The PubMed, Embase, and Cochrane databases were searched up to May 17, 2023, to identify studies on prediction nomograms for EPE of PCa. The risk of bias in studies was assessed by using the Prediction model Risk Of Bias ASsessment Tool (PROBAST). Summary estimates of sensitivity and specificity were obtained with bivariate random-effects model. Heterogeneity was investigated through meta-regression and subgroup analysis.

**Results:**

Forty-eight studies with a total of 57 contingency tables and 20,395 patients were included. No significant publication bias was observed for either the MRI-inclusive nomograms or clinical nomograms. For MRI-inclusive nomograms predicting EPE, the pooled AUC of validation cohorts was 0.80 (95% CI: 0.76, 0.83). For traditional clinical nomograms predicting EPE, the pooled AUCs of the Partin table and Memorial Sloan Kettering Cancer Center (MSKCC) nomogram were 0.72 (95% CI: 0.68, 0.76) and 0.79 (95% CI: 0.75, 0.82), respectively.

**Conclusion:**

Preoperative risk stratification is essential for PCa patients; both MRI-inclusive nomograms and traditional clinical nomograms had moderate diagnostic performance for predicting EPE in PCa. This study provides baseline comparative values for EPE prediction for future studies which is useful for evaluating preoperative risk stratification in PCa patients.

**Critical relevance statement:**

This meta-analysis firstly evaluated the diagnostic performance of preoperative MRI-inclusive nomograms and clinical nomograms for predicting extraprostatic extension (EPE) in prostate cancer (PCa) (moderate AUCs: 0.72–0.80). We provide baseline estimates for EPE prediction, these findings will be useful in assessing preoperative risk stratification of PCa patients.

**Key points:**

• MRI-inclusive nomograms and traditional clinical nomograms had moderate AUCs (0.72–0.80) for predicting EPE.

• MRI combined clinical nomogram may improve diagnostic accuracy of MRI alone for EPE prediction.

• MSKCC nomogram had a higher specificity than Partin table for predicting EPE.

• This meta-analysis provided baseline and comparative estimates of nomograms for EPE prediction for future studies.

**Graphical Abstract:**

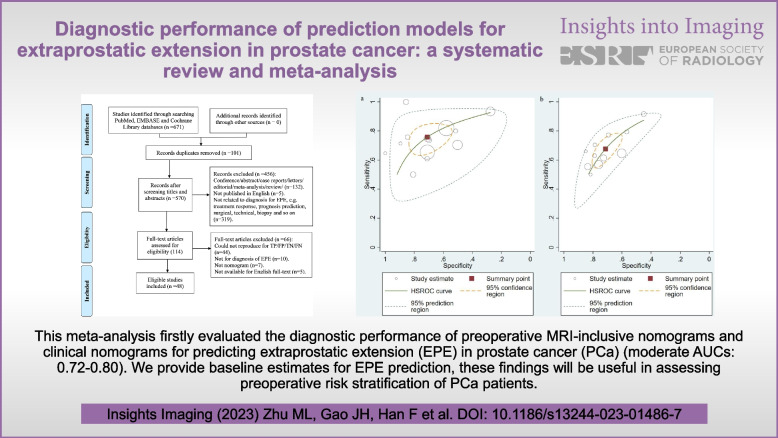

**Supplementary Information:**

The online version contains supplementary material available at 10.1186/s13244-023-01486-7.

## Introduction

Oncologic radicality and good functional outcomes are two clinical priorities for the surgical treatment of prostate cancer patients. Nerve-sparing surgery could be chosen to preserve urinary continence and erectile function for low-risk patients. However, nerve-sparing surgery in high- or moderate-risk patients is associated with an increased risk of positive surgical margins (PSMs) in postoperative specimens. Therefore, planning the therapeutic schedule for PCa patients must be based on comprehensive risk assessment and staging. Making these clinical decisions depends on the predicted probability of EPE of prostate cancer. EPE is associated with a high risk of PSMs and early biochemical recurrence and can cause a worse prognosis than organ-confined tumors [1, 2]. Consequently, accurate prediction of EPE is of high priority in clinical, radiotherapy, and surgical decision-making.

There are several widely used clinical prediction tools for evaluating EPE, such as the Partin table [3], the Cancer of the Prostate Risk Assessment (CAPRA) score [4], and the Memorial Sloan Kettering Cancer Center (MSKCC) nomogram [5]. These tools rely on conventional clinical and histopathological parameters, such as clinical T stage, prostate-specific antigen (PSA) level and biopsy Gleason score (GS). However, the diagnostic performance of these nomograms was varied, with reported areas under the curve (AUCs) ranging from 0.60 to 0.86. MRI has good tissue resolution, and some multiparametric MRI findings like capsular irregularity and bulge, curvilinear contact length (CCL) > 15 mm and invasion of periprostatic fat are associated with pathological EPE [6]. Although MRI was reported to be insensitive for diagnosing EPE (57%) by de Rooij et al., it does offer high specificity (91%) [7]. Particularly, several MRI-EPE grading systems were proposed in recent years, such as the ESUR score [8] and the EPE grade [9], which are promising to promote structure of MRI-EPE report and improve diagnostic performance. In addition, many studies have suggested that integrating MRI information with clinical characteristics might result in more precise clinical staging for PCa [10]. However, some scholars reported that MRI did not significantly increase the precision of the clinical nomogram [11, 12]. There has no well-defined MRI-inclusive nomogram to predict EPE in PCa; thus far, it is still inconclusive how to use MRI characteristics to improve the diagnostic accuracy of traditional clinical nomograms.

The previous study has summatively determined the accuracy of MRI for diagnosing EPE [7]. To our knowledge, a systematic review and meta-analysis evaluating preoperative MRI-inclusive or traditional clinical nomograms for predicting EPE has not been performed. A comprehensive systematic review is valuable for assessing the vast amount of currently available information. The purpose of this systematic review and meta-analysis is to provide baseline summative estimates for EPE prediction of PCa as well as evaluate the influence of prediction variables, to provide comparative estimates for future trial designs. We assessed study methods, adherence to reporting guidelines, and risk of bias of studies.

## Materials and methods

### Study design and search strategy

This systematic review and meta-analysis was registered with PROSPERO (CRD42022361098). This study was conducted following the Preferred Reporting Items for a Systematic Review and Meta-analysis of Diagnostic Test Accuracy Studies [13].

A systematic literature search was performed on the PubMed, Embase, and Cochrane databases up to May 17, 2023. The search terms included prostate cancer, prostate neoplasm, prostate carcinoma, extranodal extension, extraprostatic extension, extracapsular extension, nomogram, risk model, Partin table, and prediction. No language restriction was applied. We also manually reviewed the reference lists of the included studies.

### Study selection and data extraction

Two investigators (both with 6 years of research experience) independently assessed all citations according to the predefined inclusion and exclusion criteria. Disagreements were resolved through discussion and consensus. The following inclusion criteria were used: (1) primary studies for developing, validating, or updating preoperative nomograms/models (combining multiple clinical and MRI characteristics or clinical characteristics alone) to predict pathological EPE (pT3 stage) of PCa patients; and (2) studies published in English.

One investigator (with > 6 years of research experience) extracted the characteristics of all included studies independently, including study type; country; reference standards; patient age; sample size; clinical and MRI predictors; and TP, FN, TN, and FP values. To reduce the risk of data duplication and overlapping cohorts, for studies that reported multiple readers, we only extracted the results of reader 1 as a representative and incorporated them into the meta-analysis. For studies that reported multiple sensitivities/specificities of the same cohort based on different algorithms, we only extracted the highest sensitivity/specificity. For studies that reported multiple sensitivities/specificities based on different cohorts or different nomograms, we extracted all of them as independent contingency table results.

### Quality and risk of bias assessment

We assessed eligible studies for adherence to reporting guidelines according to the Transparent Reporting of a Multivariable Prediction Model for Individual Prognosis or Diagnosis (TRIPOD) checklist, which consists of 37 items in 22 criteria to aid transparent reporting of studies that develop and/or validate prediction models [14]. Five items (4b: “Specify the key study dates, including start of accrual; end of accrual; and, if applicable, end of follow-up”, 5c: “Give details of treatments received, if relevant”, 11: “Provide details on how risk groups were created, if done”, 14b: “If done, report the unadjusted association between each candidate predictor and outcome”, and 22: “Give the source of funding and the role of the funders for the present study”) were omitted since they were irrelevant to the quality assessment in this review. We deleted the element “when” in Item 6a.

The Prediction model Risk Of Bias ASsessment Tool (PROBAST) [15] was used to assess the risk of bias and applicability of each study by two investigators independently. The tool includes four domains (participants, predictors, outcome, and analysis) for risk of bias assessment and three domains (participants, predictors, and outcomes) for applicability assessment, consisting of 23 signal questions in total. Any disagreement between the two investigators was resolved by a third investigator (with > 25 years of research experience), and consensus was finally reached in all domains.

### Statistical analysis

Meta-analysis was performed with the recommended bivariate random-effects meta-analysis model [16]. Statistical heterogeneity was assessed with an *I*^2^ estimate [17]. Contingency tables were used to construct hierarchical summary receiver operating characteristic (HSROC) curves to calculate pooled sensitivities and specificities [18]. Funnel plots and Egger’s test were used to identify publication bias. A statistical significance of *p* < 0.05 indicated the presence of bias. Univariate meta-regression analysis was performed to investigate potential heterogeneity from predictors of nomograms, as well as other baseline parameters, including side-specific or whole-gland pathological EPE-based, pathological EPE rate, MRI predictor and slice thickness. Meta-analysis was conducted with Stata version 15.0.

## Results

**Fig. 1:**
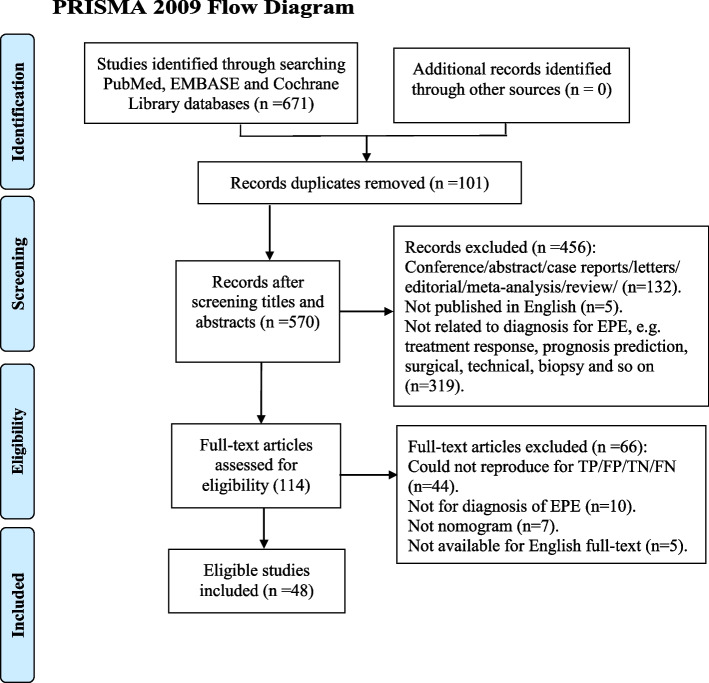
Study flow diagram

The search strategy yielded a total of 671 results (299 from Embase, 362 from PubMed, and 10 from Cochrane Library). After duplicates were removed (101), 570 studies were screened. After screening abstracts and full texts, 522 articles were excluded; the exclusion criteria are shown in Fig. 1. Finally, forty-eight full-text studies were assessed as eligible, with a total of 57 contingency tables. Twenty-two studies [9, 11, 12, 19–37] head-to-head compared the diagnostic performance of the MRI-inclusive nomogram with that of the traditional clinical nomogram but lacked data to calculate TP, FN, TN and FP; they therefore were included in the qualitative analysis only. The remaining 26 studies (13 for MRI-inclusive nomograms [29, 38–49], eight for clinical nomograms [50–57] and 5 for both MRI-inclusive nomograms and clinical nomograms [58–62]) were included in the quantitative analysis.

### Basic characteristics of the studies

A total of 20,395 patients were included. The main characteristics, demographics, and MRI information for the MRI-inclusive nomograms and clinical nomograms are presented in Tables 1 and 2, respectively. According to the TRIPOD statement (Type 1a: development only; Type 1b: development and validation using resampling; Type 2a: random split-sample development and validation; Type 3: development and validation using separate data; Type 4: validation only) [14], seventeen (35%) studies were Type 1a, eleven (23%) studies were Type 1b, four (8.3%) studies were Type 2a, one (2%) is Type 2b, six (12.5%) studies were Type 3, and nine (19%) studies were Type 4. For all included studies, the range of mean/median patient age was 61.5–70.2 years old; the range of mean/median serum PSA level was 5.75–60 ng/ml; and the range of pathological EPE rate was 0.16–0.73. Sixteen studies assessed the diagnostic performance for side-specific EPE, and the remaining 32 studies for whole-gland EPE. For quantitative analysis, three studies reported multiple different nomograms [55, 60, 61], and we only extracted the highest one into the analysis.

**Table 1:** Basic characteristics of included study for quantitative analysis (MRI-inclusive nomograms)

Author (publication year)	Study characteristics			Clinical characteristics			MRI characteristics				
	Country	TRIPOD style	No. of patients	EPE rate	Whole-gland vs. side-specific	Clinical predictors	MRI unit	Tesla	Coil	Slice thichness/gap(mm)	MRI predictor(s)
Alves 2020 [38]	Brazil	4	72	0.46	Whole-gland	biopsy GS	Philips	1.5	Body	ND	ADC, EPE on T2WI, tumor volume
Bai 2021 [39]	China	3	126	0.38	Whole-gland	PSA, biopsy GS	Siemens, GE, UIH	3	Pelvic	3–5/0–1	Peritumoral region radiomics features
Chen 2023 [48]	China	2b	304	0.38	Whole-gland	biopsy GS, number of positive cores	Siemens	3	Body	ND	Largest dimension, EPE
Giganti 2016 [40]	Italy	3	101	0.50	Whole-gland	biopsy GS	Philips	1.5	Endorectal	ND	ADC, EPE on T2WI, tumor volume
Guerra 2022 [49]	Portugal	3	228	0.26	Whole-gland	biopsy GS	Siemens	3	Pelvic	ND	Capsular disruption, CCL, EPE on MRI
Hara 2013 [41]	Tokyo	3	266	0.50	Side-specific	biopsy GS, biopsy cores ≥ 2 and maximum % of positive cores ≥ 31% on either side	Siemens	3	Body	0.73	DWI positivity
He 2021 [42]	China	2a	81	0.50	Whole-gland	biopsy GS, positive core percentage	Siemens	3	Body	3/0	Radiomics features on ADC
Hou 2021 [43]	China	3	849	0.28	Whole-gland	PSA, age, biopsy GS, % of positive cores, biopsy perineural invasion	Siemens	3	Pelvic	3.5	PAGNet model
Lebacle 2016 [44]	France	2a	1743	0.31	Whole-gland	PSA > 5 ng/ml, cT2 or T3, bGS ≥ 7, prostate weigh > 50 g	ND	ND	ND	ND	EPE stage iT3a or iT3b
Majchrzak 2021 [58]	Poland	1a	61	0.22	Side-specific	PSA, biopsy GS, maximum % of positive cores	GE or Siemens	1.5 or 3	ND	ND	EPE on MRI
Soeterik 2020 [29]	The Netherlands	4	550	0.32	Side-specific	PSA, biopsy GS, maximum % of positive cores	ND	ND	ND	ND	EPE on MRI
Wang 2018 [59]	China	1a	541	0.54	Whole-gland	Partin Table 2017	Siemens	3	Pelvic	3.5/0.3	Tumor location, MTD, PIRADS, MR stage
Xiang 2022 [62]	China	1a	105	0.42	Whole-gland	MSKCC	Siemens	1.5	ND	ND	EPE grade
Xu 2020 [45]	China	2a	128	0.38	Per-lesion	PSA, biopsy GS	GE	3	Body	3	Radiomic score
Xu 2021 [60]	China	1a	130	0.48	Whole-gland	PSAD	GE	3	Body	3	EPE grade, PIRADS
Zanelli 2019 [61]	Italy	1a	73	0.33	Whole-gland	Partin2012, MSKCC2004, CAPRA2017	Philips	3	Body	3/0	mpMRI stage
Zapała 2019 [46]	Poland	1b	88	0.30	Side-specific	PSA ≥ 20, % of positive cores ≥ 15%	Philips	1.5	Endorectal	ND	MTD ≥ 15 mm
Zapała 2021 [47]	Poland	4	154	0.23	Side-specific	PSA ≥ 20, % of positive cores ≥ 15%	Philips	3	Non-endorectal	ND	MTD ≥ 15 mm

*EPE* extraprostatic extension, *MTD* maximum tumor diameter, *GS* Gleason Score, *mpMRI* multiparametric magnetic resonance imaging, *cT* clinical tumor stage, *ND* not described, *MSKCCn* Memorial Sloan Kettering Cancer Center nomogram, *CAPRA* Cancer of the Prostate Risk Assessment

**Table 2:** Basic characteristics of included studies for quantitative analysis (clinical nomograms)

Author (publication year)	Country	TRIPOD style	No	EPE rate	Whole-gland vs. side-specific	Nomogram name	Clinical variables
Boyce 2013 [50]	Ireland	4	603	0.30	Whole-gland	ND	PSA, cT, biopsy GS
DalMoro1 2018 [51]	Italy	4	94	0.58	Side-specific	Partin 1997	PSA, cT, biopsy GS
Egawa 1998 [52]	Japan	1a	81	0.43	Whole-gland	ND	PSA, cT, biopsy GS, number of cancer cores, maximum cancer length in biopsy cores
Majchrzak 2021 [58]	Poland	4	61	0.31	Side-specific	MSKCC	PSA, cT, biopsy GS, % positive cores, % Ca
Patin 1997 [56]	USA	1b	4133	0.40	Whole-gland	Partin 1997	PSA, cT, biopsy GS
Sighinolfi 2023 [57]	Italy	4	141	0.47	Side-specific	PRECE nomogram [63]	Age, PSA, cT, GS, % positive cores
Song 2004 [54]	Korean	4	317	0.40	Whole-gland	Partin 1997	PSA, cT, biopsy GS
Thalgott 2018 [55]	Germany	4	73	0.73	Whole-gland	MSKCC, Partin 2013	PSA, cT, bGS, % positive cores, % Ca
Tsao 2014 [53]	China	4	299	0.36	Whole-gland	Partin 2007	PSA, cT, biopsy GS sum
Wang 2018 [59]	China	4	541	0.54	Whole-gland	Partin 2017	PSA, cT, biopsy GS
Xiang 2022 [62]	China	4	105	0.42	Whole-gland	MSKCC, Partin	PSA, cT, bGS, % positive cores, % Ca
Xu 2021 [60]	China	4	130	0.48	Whole-gland	CAPRA 2005, MSKCC	age, PSA, cT, primary and secondary GS, positive cores ratio
Zanelli 2019 [61]	Italy	4	73	0.33	Whole-gland	Partin 2013, MSKCC, CAPRA 2017	age, PSA, cT, primary and secondary GS, positive cores ratio

*EPE* extraprostatic extension, *GS* Gleason Score, *cT* clinical tumor stage, *ND* not described, *MSKCCn* Memorial Sloan Kettering Cancer Center nomogram, *CAPRA* Cancer of the Prostate Risk Assessment

### Studies quality assessment

**Fig. 2:**
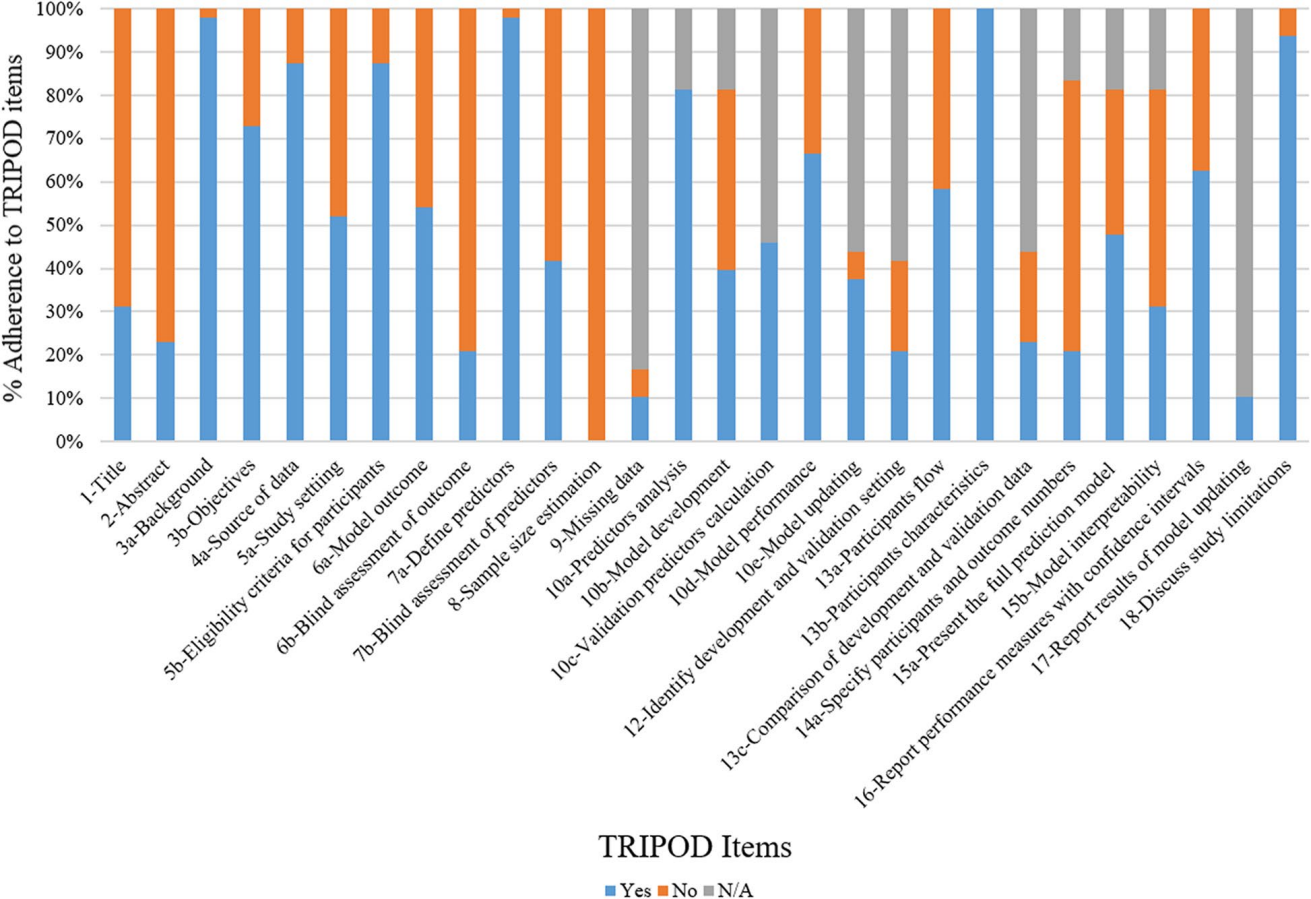
Summary of study adherence to Transparent Reporting of a Multivariable Prediction Model for Individual Prognosis or Diagnosis (TRIPOD) reporting guidelines

According to TRIPOD items (Fig. 2), the total adherence rate was 64.7% (857/1325) after excluding 290 inapplicable items. None of the studies met Item 8 (sample size estimation). In addition, 5 items were poorly reported (< 50% adherence): title (Item 1), abstract (Item 2), blind assessment of outcome (6b), blind assessment of predictors (Item 7b), specify participants and outcome numbers (Item 14a), and availability of supplementary resources (Item 21).

**Fig. 3:**
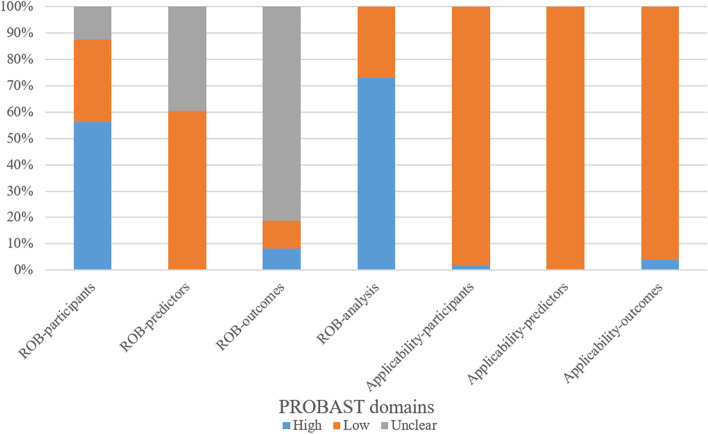
Summary of Prediction Model Study Risk of Bias Assessment Tool (PROBAST) for risk of bias and concerns of applicability

The risk of bias and applicability concerns of all studies were assessed by PROBAST (Fig. 3). For the 3 domains of participants, outcomes and analysis, there were 27 (56.3%), 4 (8.3%), and 35 (72.9%) studies had a high risk of bias, respectively. The main contributing factors to this assessment were as follows: (1) the data source of 27 (56.3%) studies was a retrospective cohort, and 6 studies did not report; (2) nineteen (39.6%) studies lacked information on whether predictor assessment was made without knowledge of outcome data; (3) four studies (8.3%) determined outcomes with knowledge of predictor(s), and most studies did not report information on outcomes determined or did not report the time interval between predictor assessment and outcome determination; and (4) the sample sizes of 18 (37.5%) studies were unreasonable (events per variable < 20), sixteen studies used univariable analysis, and 20 (41.7%) studies did not perform calibration assessment or internal validation.

### Qualitative analysis results

A summary of the qualitative analysis is presented in Supplementary Table S1, a total of 22 studies head-to-head compared the diagnostic performance of the MRI-inclusive nomogram with that of the traditional clinical nomogram. The ranges of AUCs for MRI-inclusive nomograms and clinical nomograms were 0.62–0.94 and 0.59–0.86, respectively. Twelve of the 22 studies showed that the MRI-inclusive nomogram significantly outperformed the traditional clinical nomogram (all *p* < 0.05). Two studies showed that the MRI + MSKCC/Partin nomogram provided no additional risk discrimination over the clinical nomogram alone (*p* > 0.05) [11, 12]. The remaining 8 studies also suggested that the AUC increased when MRI was added to clinical nomograms, but did not provide statistical significance.

### Main statistical analysis results

**Fig. 4:**
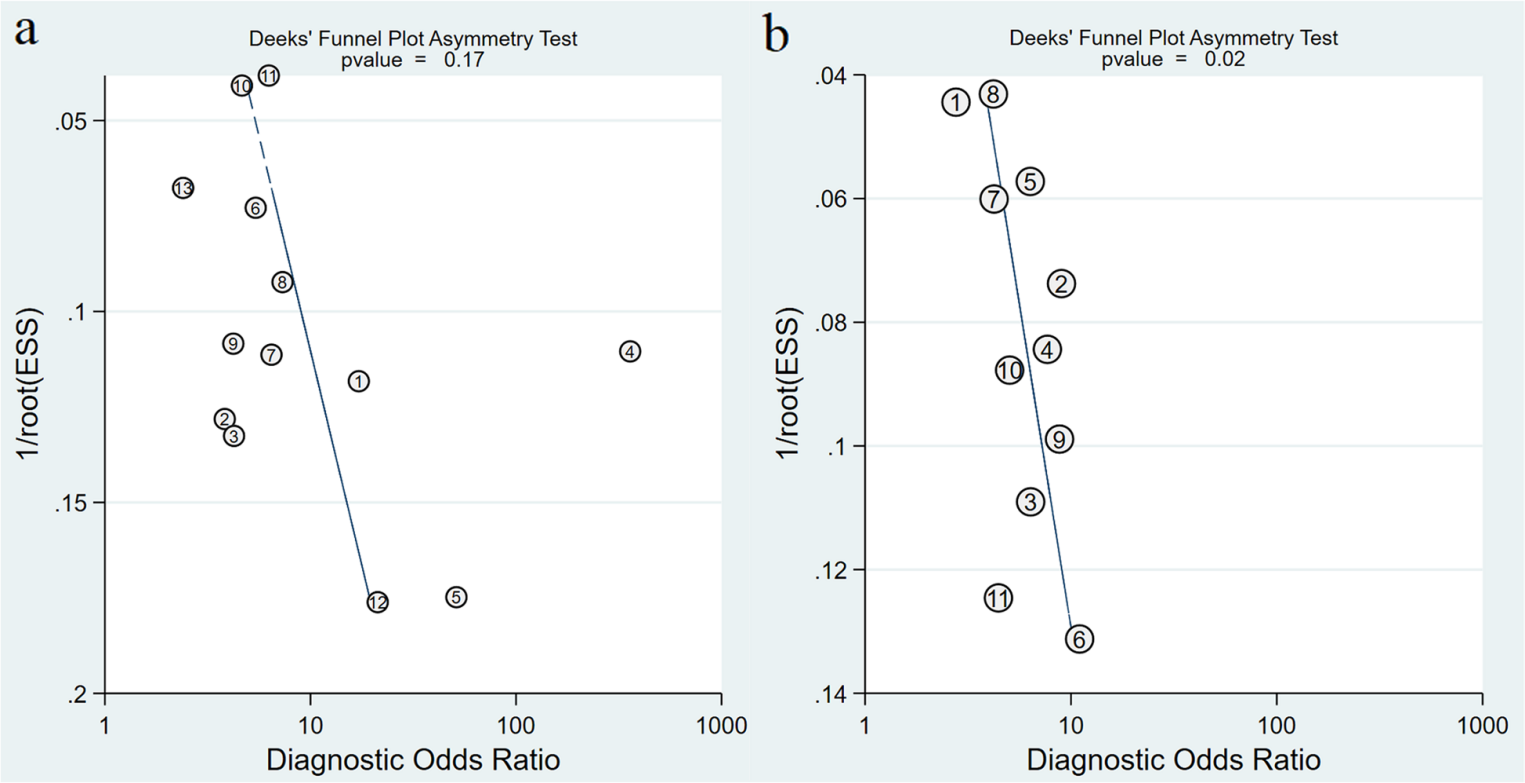
The funnel plots of publication bias for (**a**) MRI-inclusive nomograms and (**b**) traditional clinical nomograms (validation cohorts only)

**Fig. 5:**
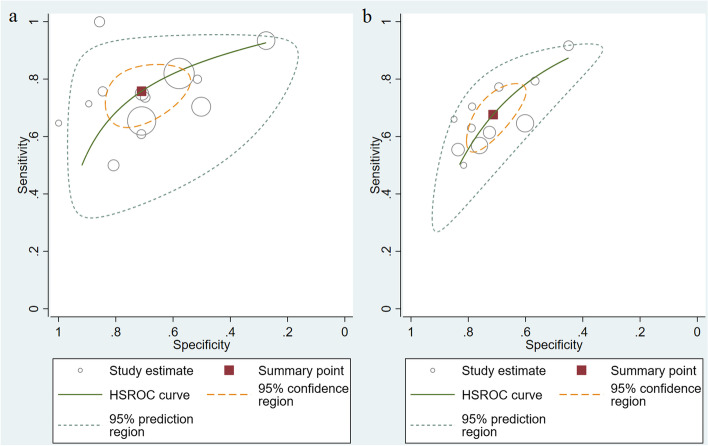
Hierarchical summary receiver operating characteristic (HSROC) curves for (**a**) MRI-inclusive nomograms on validation cohorts and (**b**) clinical nomograms on validation cohorts

Considering overfitting of performance in development models commonly existed, we therefore included validation cohorts only into meta-analysis in order to reduce overoptimism estimates. As shown in Table 3, for MRI-inclusive nomograms, a total of 13 validation cohorts [29, 38–45, 47, 48, 64] showed a pooled AUC of 0.80 (95% CI: 0.76, 0.83) for EPE prediction. For clinical nomograms, a total of 11 independent validation cohorts showed a pooled AUC of 0.75 (95% CI: 0.71, 0.79). No significant funnel plot asymmetry was observed for studies with MRI-inclusive nomogram (Fig. 4a, *P*= 0.17); however, significant funnel plot asymmetry was observed for studies with clinical nomogram (Fig. 4b, *P*= 0.02). The pooled sensitivities, specificities, and AUCs estimated by the HSROC curve (Fig. 5) of MRI-inclusive and clinical nomograms for predicting EPE are shown in Table 3. The forest plots of validation cohorts for MRI-inclusive and clinical nomograms are presented in Supplementary Figures S1- S4. In the subgroup analysis, eight external validations of the Partin table [50, 51, 53–55, 59, 61, 62] showed a pooled AUC of 0.72(95% CI: 0.68, 0.76), and 5 external validations of the MSKCC nomogram [55, 58, 60–62] showed a pooled AUC of 0.79(95% CI: 0.75, 0.82).

**Table 3:** Pooled sensitivities, specificities, and AUCs for MRI-inclusive and clinical nomograms for predicting EPE (validation cohorts only)

Nomogram	No. of cohorts	No. of patients	Sensitivity (95% CI)	Heterogeneity		Specificity (95% CI)	Heterogeneity		AUC (95% CI)
				*I* ^2^ (%)	Cochran *Q p* value		*I*^2^ (%)	Cochran *Q p* value	
MRI-inclusive nomograms	13	2177	0.76 (0.67, 0.83)	81.18 (71.69, 90.66)	< 0.001	0.71 (0.59, 0.80)	94.75 (92.92, 96.58)	< 0.001	0.80 (0.76, 0.83)
Clinical nomograms	11	2437	0.68 (0.59, 0.75)	84.21 (75.93, 92.49)	< 0.001	0.71 (0.64, 0.78)	88.50 (82.98, 94.02)	< 0.001	0.75 (0.71, 0.79)
Partin table	8	2105	0.73 (0.62, 0.82)	88.29 (82.57, 95.01)	< 0.001	0.60 (0.47, 0.72)	93.26 (89.97, 96.56)	< 0.001	0.72 (0.68, 0.76)
MSKCCn	5	442	0.68 (0.60, 0.75)	26.26 (0, 94.20)	0.25	0.76 (0.70, 0.82)	28.18 (0, 95.54)	0.23	0.79 (0.75, 0.82)

*CI* confidence interval, *AUC* area under the receiver operating characteristic curve, *MSKCCn* Memorial Sloan Kettering Cancer Center nomogram

### Univariable meta-regression analysis

Meta-regression analysis was performed for all validation cohorts to identify the source of pooled heterogeneity (Table 4). For MRI-inclusive nomograms, the heterogeneity of pooled sensitivity was found in EPE-basing and AI/radiomics-based, and the sensitivity of nomograms which predicted side-specific EPE was higher than predicting whole-gland EPE (*p* = 0.01). The source of heterogeneity of pooled specificity did not be found from EPE basing, pathological EPE rate, slice thickness, MRI time, or AI-based predictors (all *p* > 0.05). For clinical nomograms, the pooled specificity of the MSKCC nomogram was significantly higher than that of the Partin table (*p* < 0.001); there was no significant difference of pooled sensitivity between the Partin table and MSKCC nomogram.

**Table 4:** Univariable meta-regression evaluating the effect of confounding factors on sensitivities and specificities of MRI-inclusive nomograms and clinical nomograms for EPE prediction

Parameter	Category	No. of cohorts	Sensitivity (95% CI)	*p* value	Specificity (95% CI)	*p* value
MRI-inclusive nomograms						
EPE based on	Whole-gland	9	0.73 (0.64–0.82)	**0.01**	0.76 (0.68–0.85)	0.42
	Side-specific	4	0.81 (0.71–0.92)		0.54 (0.37–0.71)	
pEPE rate	≥ 0.4	5	0.74 (0.61–0.87)	0.09	0.81 (0.67–0.94)	0.82
	< 0.4	8	0.77 (0.68–0.86)		0.65 (0.52–0.78)	
MRI time	Before biopsy	4	0.72 (0.59–0.85)	0.09	0.72 (0.49–0.95)	0.97
	After biopsy	4	0.77 (0.66–0.88)		0.68 (0.42–0.94)	
Slice thickness	> 3 mm	4	0.68 (0.52–0.84)	0.07	0.70 (0.53–0.87)	0.84
	≤ 3 mm	3	0.82 (0.68–0.95)		0.61 (0.37–0.84)	
AI/radiomics-based	Yes	6	0.69 (0.57–0.82)	**0.01**	0.73 (0.59–0.88)	0.60
	No	7	0.80 (0.71–0.88)		0.69 (0.54–0.84)	
Clinical nomograms						
Model	Partin Table	8	0.73 (0.64–0.81)	0.26	0.60 (0.50–0.70)	** < 0.001**
	MSKCCn	5	0.68 (0.56–0.80)		0.77 (0.67–0.87)	

*EPE* extraprostatic extension, *pEPE* pathological EPE, *AI* artificial intelligence, *DCE* dynamic contrast enhancement, *MSKCCn* Memorial Sloan Kettering Cancer Center nomogram

### Evaluation of clinical utility

**Fig. 6:**
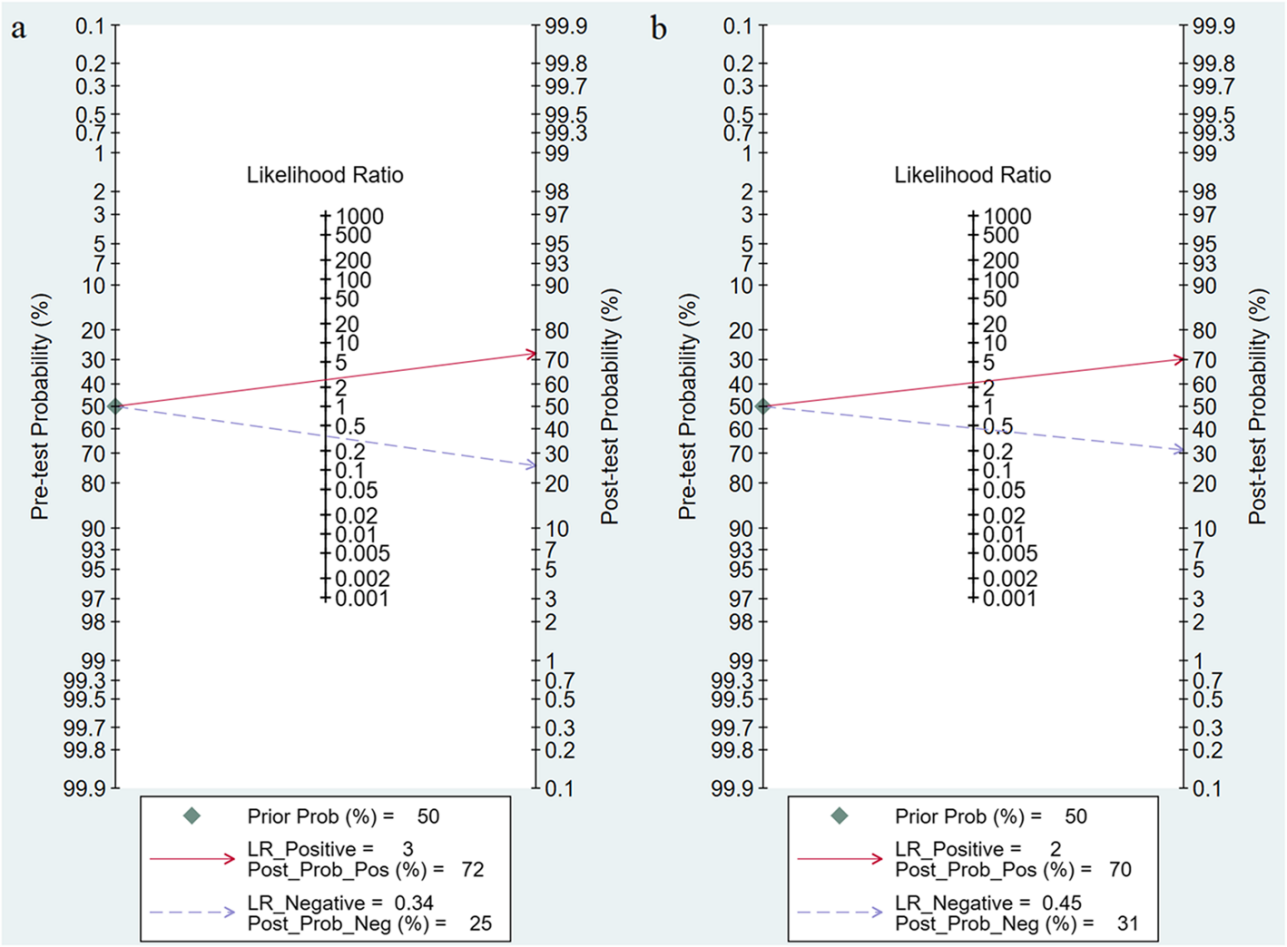
Likelihood ratios and posttest probabilities for (**a**) MRI-inclusive nomograms and (**b**) clinical nomograms for predicting EPE (validation cohorts only)

Fagan plots were drawn to calculate the posttest probabilities of all validation cohorts for MRI-inclusive nomogram and clinical nomogram respectively (Fig. 6). Within 50% as the pretest probability, for MRI-inclusive nomograms and clinical nomograms, the positive posttest probabilities were 72% and 70%, respectively; the negative posttest probabilities were 25% and 31%, respectively; and the positive likelihood ratios were 3 and 2, respectively.

## Discussion

This present meta-analysis first systematically evaluated the diagnostic performance of preoperative MRI-inclusive nomograms and traditional clinical nomograms for predicting pathological EPE of PCa. Our meta-analysis based on validation cohorts showed that both MRI-inclusive nomograms and traditional clinical nomograms had moderate AUCs (0.72–0.80) for predicting EPE. We found that the pooled specificity of MSKCC nomograms was higher than that of Partin table. Our study provides baseline summative estimates for EPE prediction in PCa as well as provide comparative estimates for future study designs. These findings will be useful in the assessment of preoperative risk stratification and individual treatment strategies for PCa patients.

There are multitudinous studies that tried to head-to-head compare the diagnostic performance of the MRI-inclusive nomogram and traditional clinical nomogram for EPE prediction and suggested that MRI combined clinical nomogram can improve the diagnostic performance of traditional clinical nomogram. However, in our systematic review of 22 studies, we found that most of these studies were TRIPOD 1 type, which means they were limited to developing model. As we know, the overfitting of prediction model generally exists in development cohorts, particularly in a small data set. And the degree of overfitting can vary widely depending on the type of internal validation techniques employed, such as cross-validation or bootstrapping, leading to biased or over-optimistic performance results [15]. Therefore, these conclusions need to be validated in more external validation cohorts to make them more reliable. However, what predictors or how to add MRI variables into clinical nomograms was still not well-defined.

Compared with a previous meta-analysis by de Rooij et al. [7], which reported that MRI had poor and heterogeneous sensitivity (0.57) for EPE diagnosis, our results may provide evidence that MRI combined clinical nomogram had relatively higher sensitivity (0.76). Reasons for the low and variable sensitivity of MRI for EPE diagnosis may be as follows. First, image acquisition protocol can largely influence image quality. Moreover, connective tissue hyperplasia reaction or inflammation may change the appearance of prostatic capsule, subjectivity of assessing some qualitative features like bulging and irregularity of prostatic capsule could not be ruled out, and radiologic interpretation may be influenced by the specialization and experience of radiologists [65]. Thus, its accuracy for EPE diagnosis on MRI evaluation remains challenging. To overcome this issue, radiologists have been devoted to standardize the EPE reporting to improve its precision and repeatability, and several EPE grading systems have been proposed in recent years with relatively good performance [8, 9, 66]. Moreover, several scholars have reported that combined MRI-EPE grade with traditional clinical nomograms can significantly improve the diagnostic accuracy [9, 25]. Therefore, we suggested that more external validation studies for the MRI-EPE grade combined clinical nomograms should be carried on in the future research. Second, it was realized that sensitivity for detecting EPE on MRI can never match pathology, where the standard is microscopy [67]. However, few studies have focused on the prediction of microscopic EPE to date. Thus, improvements are needed for both the clinic and imaging to further investigate the predictive efficiency for detecting microscopic EPE.

In particular, although our regression analysis did not find significant diagnostic superiority of nomogram which using AI/radiomics-based MRI predictors in EPE prediction, this may be due to the limited statistic power given the small number of studies on AI/radiomics-based MRI predictors (6 contingency tables from 4 studies [39, 42, 43, 45]). Moreover, the AI/radiomics-based MRI predictors of these 4 studies were heterogeneous, involving peritumoral region radiomic features [39], tumor radiomic signatures extracted from ADC [42], the ResNeXt network model [43], and the self-defined radiomic score [45]. Thus, more studies are needed to evaluate the optimal and repeatable radiomic characteristics of multiple MR sequences for EPE diagnosis. Many studies have proposed that artificial intelligence, including radiomics and deep learning, is a promising solution to diagnosis and stage PCa [68–70]. However, the combination of AI-based MRI characteristics and clinical variables to predict EPE has not yet been fully explored.

In our meta-regression analysis, no significant difference in sensitivity was observed between the Partin tables and MSKCC nomogram. The specificity of the MSKCC nomogram was significantly higher than that of the Partin table (0.77 vs. 0.60). One difference between the MSKCC nomogram and the Partin table is the addition of the biopsy-positive core ratio. It has been reported that using continuous risk variables in nomograms rather than binary variables can substantially improve predictive accuracy [71], which may be a probable explanation for why the MSKCC nomogram had a better diagnostic performance. In general, the predictive accuracy for EPE of all clinical nomograms was relatively low and diverse (AUC: 0.60–0.86). Several molecular markers have been discovered for predicting EPE (e.g., interleukin-6 soluble receptor, transforming growth factor-b1, GRE), incorporating these new markers into traditional nomograms may improve the prediction ability of disease progression [71, 72]. Therefore, in the era of PSA screening, as the composition of patients with localized disease increases, nomograms need to be periodically updated, and new biomarkers need to be added to reflect these population changes and to improve the prediction accuracy [73].

Some limitations existed in our study. First, the number of eligible studies included was small, and a large number of studies were excluded due to a lack of data to calculate TP, FN, FP, and TN. Second, there was significant heterogeneity in patient populations, clinical characteristics, and MRI practice, which increased the risk of intrinsic bias and led to significant heterogeneity (*I*^2^). Third, we could not explain the heterogeneity completely because many studies did not report sufficient information for all characteristics. Finally, there are no established MRI assessment criteria for EPE; therefore, the imaging predictors varied widely. To overcome these issues, we suggest that studies should be devoted to establishing a standardized MRI-EPE evaluation system, which would be helpful to improve diagnostic accuracy and repeatability. Moreover, nomograms need to be updated, and new biomarkers need to be added to further improve the diagnostic performance. In addition, no studies have focused on predicting microscopic EPE, which is the most relevant for RP purposes, as overt EPE is presumably easily found on MRI; thus, more studies should be carried out to evaluate microscopic EPE in the future.

## Conclusion

This systematic review and meta-analysis first summatively evaluated the diagnostic accuracy of preoperative MRI-inclusive nomograms and traditional clinical nomograms in predicting pathological EPE of PCa. Both MRI-inclusive nomograms and traditional clinical nomograms had moderate AUCs (0.72–0.80) for predicting EPE. MRI combined clinical predictors can improve diagnostic value to MRI alone, which could aid urologists in making decision protocols for local PCa patient treatment.

### Supplementary Information

**Additional file 1.** Supplementary material. Supplementary Figures. Supplementary Tables.

## Abbreviations

AUC: Area under the curve
CAPRA: Cancer of the Prostate Risk Assessment
EPE: Extraprostatic extension
FN: False negative
FP: False positive
HSROC: Hierarchical summary receiver operating characteristic
MSKCC: Memorial Sloan Kettering Cancer Center
PCa: Prostate cancer
TN: True negative
TP: True positive
