# Supplementary material for: Diagnostic performance of prediction models for extraprostatic extension in prostate cancer: a systematic review and meta-analysis
Source: Insights Imaging. 2023 Aug 22;14:140. doi: 10.1186/s13244-023-01486-7 (PMC10444717; doi:10.1186/s13244-023-01486-7)
Supplement: Supplementary file 1 — Additional file 1. Supplementary material. Supplementary Figures. Supplementary Tables. [file 13244_2023_1486_MOESM1_ESM.pdf]

Diagnostic performance of prediction models for extraprostatic extension in prostate cancer: a systematic review and meta-analysis

ELECTRONIC SUPPLEMENTARY MATERIAL

Supplementary Material
Search strategies

Database: EMBASE
Search strategies run on 17/05/2023 by Meilin Zhu.
#1 ‘prostate cancer’/exp
#2 ‘prostate carcinoma’:ab,ti
#3 #1 OR #2 (248094)
#4 ‘Extranodal Extension’ /exp
#5 ‘Extranodal Extension’:ab,ti
#6 ‘Extracapsular Extension’:ab,ti
#7 #4 OR #5 OR #6 (3140)
#8 ‘Nomogram’ /exp
#9 ‘Nomograms’:ab,ti
#10 ‘risk model’ /exp
#11 ‘prediction’ /exp
#12 #8 OR #9 OR #10 OR #11 (466154)
#13 #3 AND #7 AND #12 AND (299)

Database: PubMed
Search strategies run on 16/05/2023 by Meilin Zhu.
#1 Prostatic Neoplasms [MeSH Major Topic]
#2 prostatic cancer [Title/Abstract]
#3 prostate cancer [Title/Abstract]
#4 prostate carcinoma [Title/Abstract]
#5 #1 OR #2 OR #3 OR #4 (174812)
#6 Extranodal Extension [MeSH Major Topic]
#7 Extracapsular Extension [Title/Abstract]
#8 Extraprostatic extension [Title/Abstract]
#9 EPE [Title/Abstract]
#10 ECE [Title/Abstract]
#11 #6 OR #7 OR #8 OR #9 (5058)
#12 Nomograms [MeSH Major Topic]
#13 Nomogram [Title/Abstract]
#14 Partin Table [Title/Abstract]
#15 Partin Nomogram [Title/Abstract]
#16 Risk [MeSH Major Topic]
#17 Risks [Title/Abstract]
#18 Models, Statistical [MeSH Major Topic]

#19 Model [Title/Abstract]
#20 #12 OR #13 OR #14 OR #15 OR #16 OR #17 OR #18 OR #19 (3015696)
#21 #5 AND #11 AND #20 (362)

Database: Cochrane library and CENTRAL
Search strategies run on 17/05/2023 by Meilin Zhu.
#1 Prostatic Neoplasms
#2 Prostatic cancer
#3 Prostatic carcinoma
#4 #1 OR #2 OR #3 (16327)
#5 Extranodal Extension
#6 Extracapsular Extension
#7 Extraprostatic extension
#8 #5 OR #6 OR #7 (158)
#9 Nomograms
#10 Nomogram
#11 Risk assessment
#12 #9 OR #10 OR #11 (33913)
#13 #4 AND #8 AND #12 (10)

PRISMA-DTA Checklist Item

Section/topic	#	PRISMA-DTA Checklist Item	Reported on page #
**TITLE / ABSTRACT**			
Title	1	Identify the report as a systematic review (+/- meta-analysis) of diagnostic test accuracy (DTA) studies.	1
Abstract	2	Abstract: See PRISMA-DTA for abstracts.	1
**INTRODUCTION**			
Rationale	3	Describe the rationale for the review in the context of what is already known.	2
Clinical role of index test	D1	State the scientific and clinical background, including the intended use and clinical role of the index test, and if applicable, the rationale for minimally acceptable test accuracy (or minimum difference in accuracy for comparative design).	2-3
Objectives	4	Provide an explicit statement of question(s) being addressed in terms of participants, index test(s), and target condition(s).	3
**METHODS**			
Protocol and registration	5	Indicate if a review protocol exists, if and where it can be accessed (e.g., Web address), and, if available, provide registration information including registration number.	3
Eligibility criteria	6	Specify study characteristics (participants, setting, index test(s), reference standard(s), target condition(s), and study design) and report characteristics (e.g., years considered, language, publication status) used as criteria for eligibility, giving rationale.	3, Figure 1
Information sources	7	Describe all information sources (e.g., databases with dates of coverage, contact with study authors to identify additional studies) in the search and date last searched.	3-4
Search	8	Present full search strategies for all electronic databases and other sources searched, including any limits used, such that they could be repeated.	3

Study selection	9	State the process for selecting studies (i.e., screening, eligibility, included in systematic review, and, if applicable, included in the meta-analysis).	3-4
Data collection process	10	Describe method of data extraction from reports (e.g., piloted forms, independently, in duplicate) and any processes for obtaining and confirming data from investigators.	3-4, Figure 1
Definitions for data extraction	11	Provide definitions used in data extraction and classifications of target condition(s), index test(s), reference standard(s) and other characteristics (e.g. study design, clinical setting).	3-4
Risk of bias and applicability	12	Describe methods used for assessing risk of bias in individual studies and concerns regarding the applicability to the review question.	4
Diagnostic accuracy measures	13	State the principal diagnostic accuracy measure(s) reported (e.g. sensitivity, specificity) and state the unit of assessment (e.g. per-patient, per-lesion).	4
Synthesis of results	14	Describe methods of handling data, combining results of studies and describing variability between studies. This could include, but is not limited to: a) handling of multiple definitions of target condition. b) handling of multiple thresholds of test positivity, c) handling multiple index test readers, d) handling of indeterminate test results, e) grouping and comparing tests, f) handling of different reference standards	4, 5
Meta-analysis	D2	Report the statistical methods used for meta-analyses, if performed.	4
Additional analyses	16	Describe methods of additional analyses (e.g., sensitivity or subgroup analyses, meta-regression), if done, indicating which were pre-specified.	4
**RESULTS**			
Study selection	17	Provide numbers of studies screened, assessed for eligibility, included in the review (and included in meta-analysis, if applicable) with reasons for exclusions at each stage, ideally with a flow diagram.	4-5
Study characteristics	18	For each included study provide citations and present key characteristics including: a) participant characteristics (presentation, prior testing), b) clinical setting, c) study design, d)target condition definition, e) index test, f) reference standard, g) sample size, h) funding sources	5, Table 1,2 and Table S2

Risk of bias and applicability	19	Present evaluation of risk of bias and concerns regarding applicability for each study.	5, Figure 2 and 3
Results of individual studies	20	For each analysis in each study (e.g. unique combination of index test, reference standard, and positivity threshold) report 2x2 data (TP, FP, FN, TN) with estimates of diagnostic accuracy and confidence intervals, ideally with a forest or receiver operator characteristic (ROC) plot.	Table 3, Figures 5, Figures S1-4
Synthesis of results	21	Describe test accuracy, including variability; if meta-analysis was done, include results and confidence intervals.	5, Table 3
Additional analysis	23	Give results of additional analyses, if done (e.g., sensitivity or subgroup analyses, meta-regression; analysis of index test: failure rates, proportion of inconclusive results, adverse events).	5-6, Table 4
**DISCUSSION**			
Summary of evidence	24	Summarize the main findings including the strength of evidence.	6
Limitations	25	Discuss limitations from included studies (e.g. risk of bias and concerns regarding applicability) and from the review process (e.g. incomplete retrieval of identified research).	9
Conclusions	26	Provide a general interpretation of the results in the context of other evidence. Discuss implications for future research and clinical practice (e.g. the intended use and clinical role of the index test).	9-10
**FUNDING**			
Funding	27	For the systematic review, describe the sources of funding and other support and the role of the funders.	10

Adapted From: McInnes MDF, Moher D, Thombs BD, McGrath TA, Bossuyt PM, The PRISMA-DTA Group (2018). Preferred Reporting Items for a Systematic Review and Meta-analysis of Diagnostic Test Accuracy Studies: The PRISMA-DTA Statement. JAMA. 2018 Jan 23;319(4):388-396. doi: 10.1001/jama.2017.19163.

Table S1: Basic characteristics of included studies for qualitative analysis (MRI combined clinical nomograms)										
Author	Country	TRIPOD style	No.	EPE rate	Whole-gland vs. Side-Specific	MRI nomogram/predictors	Clinical nomogram/predictors	AUC (MRI+clinical vs. clinical)	*P*	Main findings
Blas 2023 [35]	Japan	4	178	0.33	Side-specific	Soeterik model [29], Martini model [23]	MSKCC, Partin, Patel[73]	0.75-0.81 vs. 0.60-0.78	ND	The best-performing nomograms were the Soeterik and the Patel models
Chen 2017 [19]	China	1b	353	0.56	Side-specific	ESUR EPE score	MSKCC 2006	0.851 vs. 0.796	0.003	MSKCC was significantly inferior to the MRI added model.
Diamand 2021 [20]	European centers*	4	566	0.37	Whole-gland	MTD, positive/negative EPE	MSKCC, Partin 2013	0.718 vs. 0.698, 0.718 vs. 0.613	0.3, <0.001	The nomogram showed higher discrimination.
Diamand 2023 [36]	European centers*	4	737	0.29	Side-specific	PSAD, ECE on MRI	PSA, cT stage, ISUP GG, % positive cores			MRI combined with clinicobiochemical parameters achieved the highest model performance
Feng 2015 [21]	USA	1a	112	0.23	Whole-gland	positive/negative EPE	MSKCC 2004, Partin 2013	0.94 vs. 0.86, 0.93 vs. 0.85	0.023, 0.017	AUC increased when MRI was added to each nomogram.
Gandaglia 2019 [32]	European centers*	1b	614	0.54	Whole-gland	MTD, positive/negative EPE	PSA, biopsy GS, % of cancer cores	0.73 vs. 0.67	ND	Inclusion MRI data improved the

										discrimination of clinical models.
Jansen 2019 [11]	Netherlands	1a	430	0.32	Whole-gland	MRI T-stage	MSKCC 2003, Partin 2017	0.74 vs. 0.73, 0.66 vs. 0.62	ND	Addition MRI did not increase diagnostic accuracy.
Liu 2020 [33]	China	1a	222	0.37	Whole-gland	MTD, positive/negative EPE	PSA, PSAD, biopsy GS, No. of positive biopsy cores	0.842 vs 0.766	ND	Model including clinical and MRI data achieved the highest AUC.
Losnega°rd 2020 [22]	Norway	1b	228	0.38	Whole-gland	MR radiomic features, Mehralivand EPE grade	MSKCC 2018	0.80 vs. 0,67	<0.05	The combination model gave the highest AUC.
Martini 2018 [23]	Italy	1b	561	0.17	Side-specific	positive/negative EPE	PSA, biopsy GS, maximum % core	0.83 vs. ND	ND	MRI improved clinical risk prediction of EPE.
Mehralivand 2019 [9]	Germany	1b	553	0.22	Whole-gland	The EPE grade	PSA, biopsy ISUP stage	0.81 vs. 0.71	<0.001	The AUC improved when combining the MRI-EPE grade with clinical parameters.
Morlacco 2016 [24]	USA	1a	501	0.42	Whole-gland	positive/negative EPE	Partin 2013, CAPRA 2005	0.73 vs. 0.61, 0.77 vs. 0.69	ND	MRI+clinical models outperformed clinical models.
Nyarangi-Dix 2020 [25]	Germany	1b	264	0.48	Side-specific	ESUR score, capsule contact length, tumor volume	MSKCC 2018	0.85 vs. 0.73	<0.001	EPE-RM model was significantly better than clinical model.

Ravi 2021 [26]	Indian	1b	273	0.50	Whole-gland	positive/negative EPE	Partin 2013	0.826 vs. 0.67	<0.001	The new nomogram has higher predictive accuracy compared to Partin Table.
Rayn 2018 [27]	Germany	1a	532	0.22	Whole-gland	NIH suspicion score, EPE, MTD	Partin 2011, MSKCC 2018	0.80 vs. 0.66, 0.80 vs. 0.70	<0.001 0.003	MRI+clinical nomograms provide significant additional predictive ability.
Renard-Penna 2011 [28]	France	1a	101	0.16	Whole-gland	positive/negative EPE	DRE, PSA, biopsy GS	0.895 vs. 0.758	ND	The accuracy of combined MRI was significantly higher than that of clinical model alone.
Sandeman 2020 [34]	Finland	1a	387	0.43	Whole-gland	EPE, PIRADS≥3, prostate volume	Partin 2013, MSKCC 2011	0.62 vs. 0.73, 0.71 vs. 0.78	<0.001 <0.001	Combing MRI improves the predictive value of clinical models.
Soeterik 2020 [29]	The Netherlands	3	887	0.38	Side-specific	positive/negative EPE	cT, biopsy GS, % of positive cores	0.82 vs. 0.80	ND	MRI-inclusive models resulted higher AUC than models without MRI.
Sun 2022 [37]	China	1a	152	0.53	Whole-gland	EPE on MRI, PI-RADS ≥4	PSA, biopsy ISUP	0.85 vs. 0.79	0.031	Inclusion of mp-MRI improved discrimination by clinical models for EPE

Wang 2004 [30]	USA	1a	34 4	0.17	Whole-gland	five-point scale	PSA, % of cancer cores	0.838 vs. 0.772	0.022	MRI findings add incremental value.
Weaver 2017 [12]	USA	1a	23 7	0.35	Whole-gland	positive/negative EPE	MSKCC 2004	0.77 vs. 0.74	0.079	MRI+MSKCC nomogram provides no additional risk discrimination over the MSKCC nomogram.
Wibmer 2021 [31]	USA, France Germany, Denmark, Italy, Spain	1b	84 0	0.32	Side-specific	PI-RADS, positive/negative EPE, capsule contact length	MSKCC 2019, Partin 2018	0.828 vs. 0.675, 0.828 vs. 0.601	<0.00 1	MRI-inclusive nomogram has significantly greater accuracy than clinical benchmark models.

Note.—EPE=extraprostatic extension, TRIPOD= Transparent Reporting of a Multivariable Prediction Model for Individual Prognosis or Diagnosis, MTD= maximum tumour diameter, GS=Gleason Score, MRI= magnetic resonance imaging, cT=clinical tumor stage, PSAD=PSA density, ND=not described, DRE=digital rectal exam, ESUR=European Society of Urogenital Radiology, MSKCCn= Memorial Sloan Kettering Cancer Center nomogram, CAPRA=Cancer of the Prostate Risk Assessment.

*Including Belgium, France, Switzerland, and Italy.

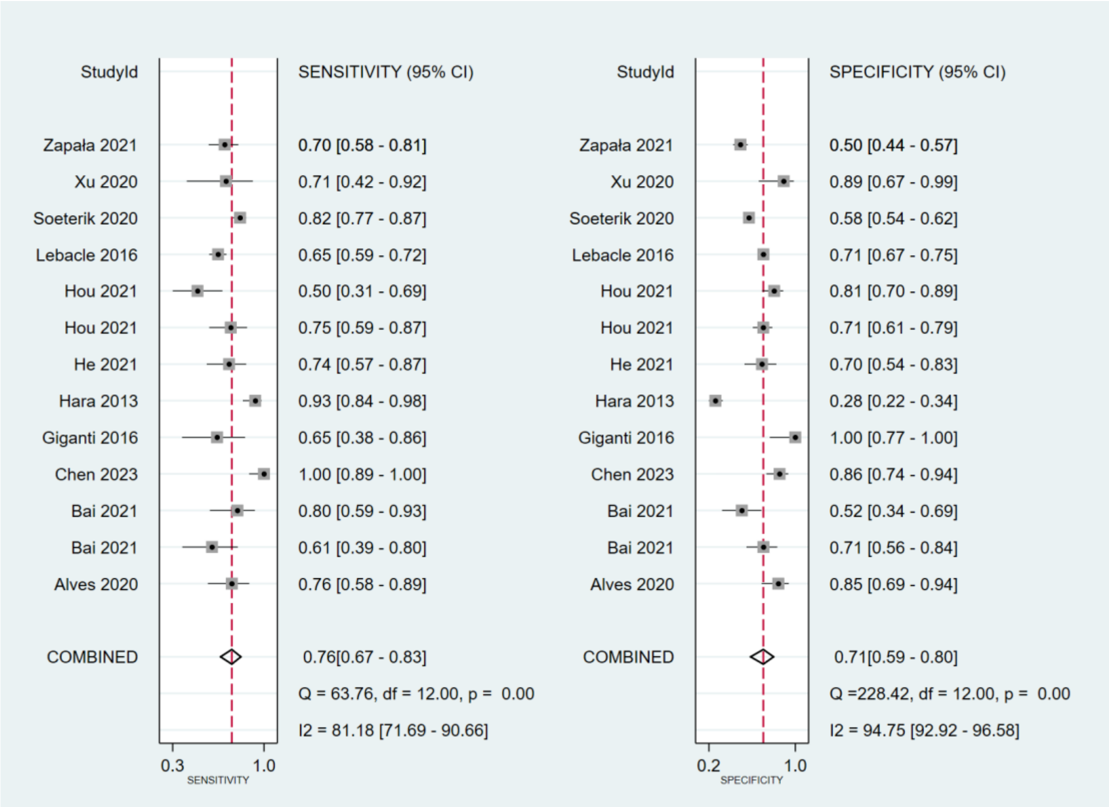

Figure S1: Forest plot of the MRI-inclusive nomograms (validation cohorts) for predicting EPE.

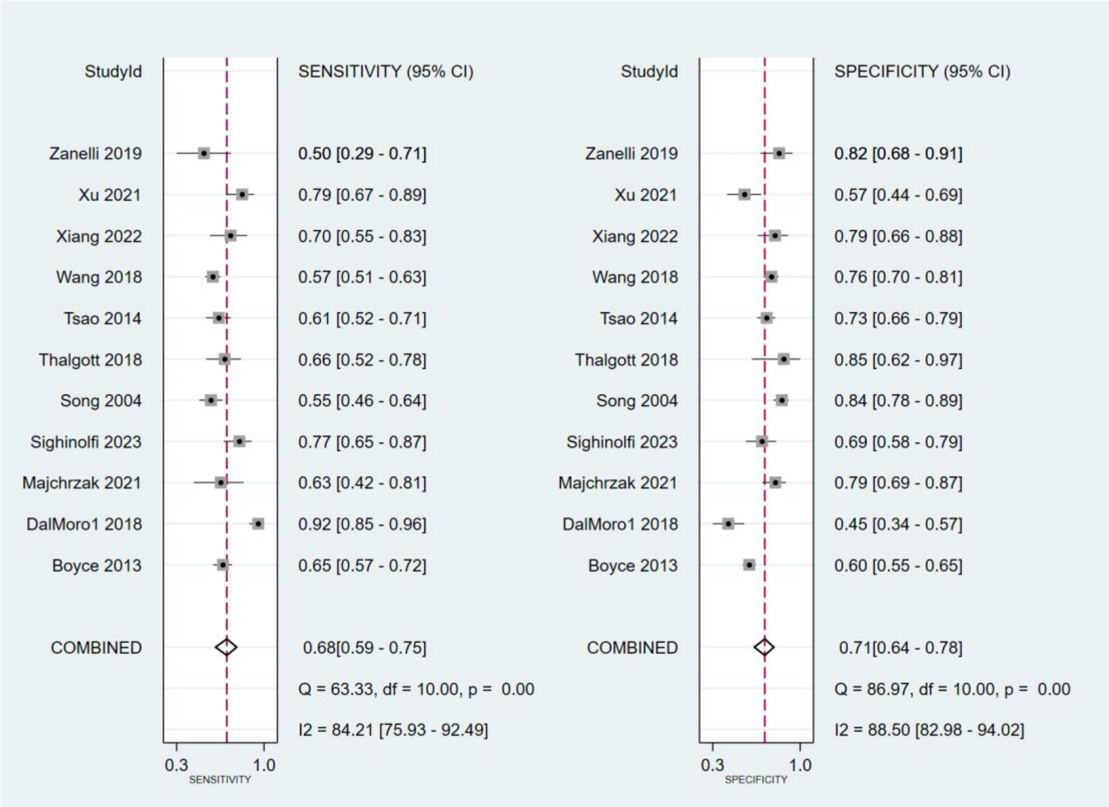

Figure S2: Forest plot of all clinical nomograms (validation cohorts) for predicting EPE.

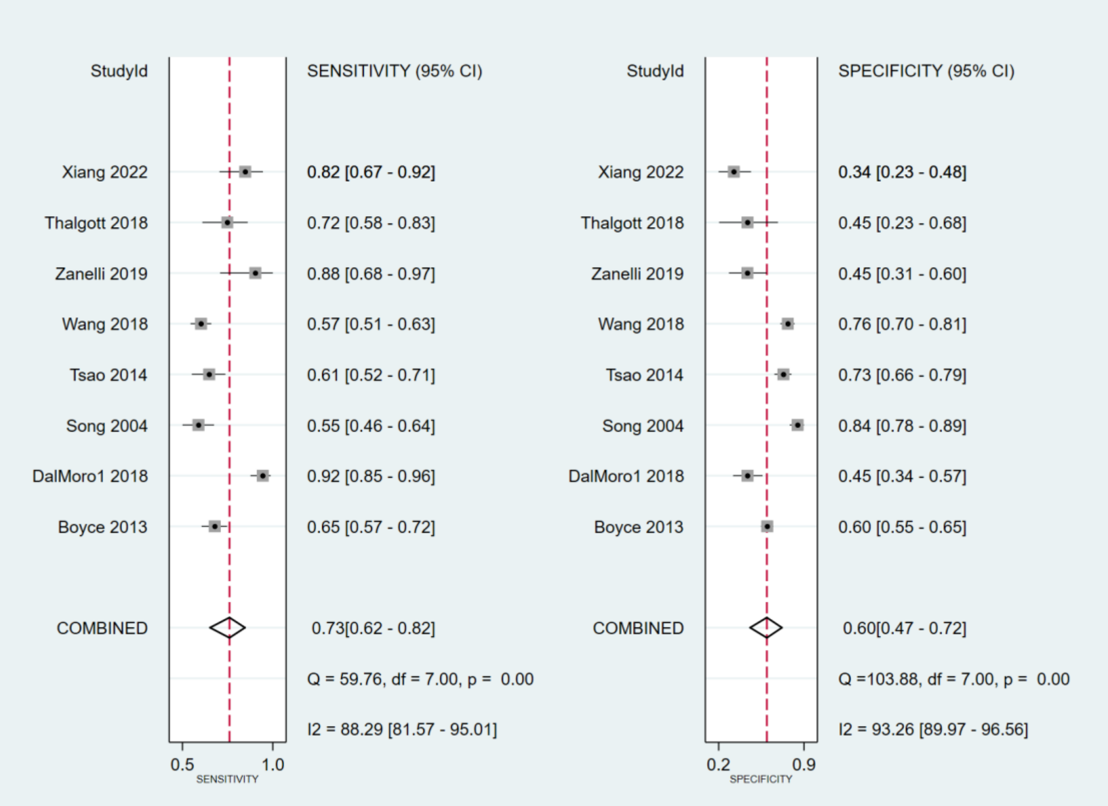

Figure S3: Forest plot of the Partin tables (validation cohorts) for predicting EPE.

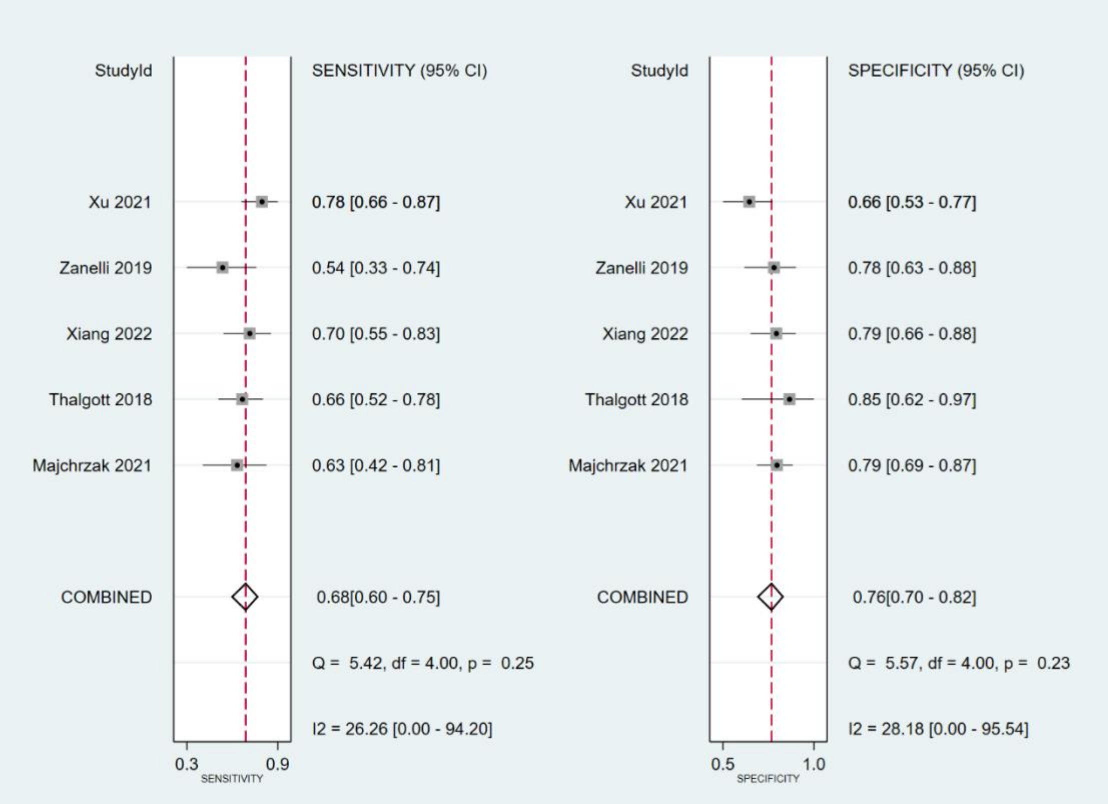

Figure S4: Forest plot of the MSKCC nomograms (validation cohorts) for predicting EPE.
